# Apache Spark and Deep Learning Models for High-Performance Network Intrusion Detection Using CSE-CIC-IDS2018

**DOI:** 10.1155/2022/3131153

**Published:** 2022-08-26

**Authors:** Abdulnaser A. Hagar, Bharti W. Gawali

**Affiliations:** ^1^Faculty of Administrative and Computer Sciences, Albaydha University, Albaydha, Yemen; ^2^Department of Computer Science and Information Technology, Dr. Babasaheb Ambedkar Marathwada University, Aurangabad, India

## Abstract

Keeping computers secure is becoming challenging as networks grow and new network-based technologies emerge. Cybercriminals' attack surface expands with the release of new internet-enabled products. As many cyberattacks affect businesses' confidentiality, availability, and integrity, network intrusion detection systems (NIDS) show an essential role. Network-based intrusion detection uses datasets like CSE-CIC-IDS2018 to train prediction models. With fourteen types of attacks included, the latest big data set for intrusion detection is available to the public. This work proposes three models, two deep learning convolutional neural networks (CNN), long short-term memory (LSTM), and Apache Spark, to improve the detection of all types of attacks. To reduce the dimensionality, random forests (RF) was employed to select the important features; it gave 19 from 84 features. The dataset is imbalanced; thus, oversampling and undersampling techniques reduce the imbalance ratio. The Apache Spark model produced the best results across all 15 classes, with accuracy as high as 100% for all classes, as seen by the experiments' findings. For the *F*1-score, Apache Spark showed the highest results with 1.00 for most classes. The findings of the three models showed outstanding results for multiclassification network intrusion detection.

## 1. Introduction

Information and computer security is a growing concern. Over the past two decades, infiltration techniques and security defenses have progressed significantly. Even though cyberattacks have developed new approaches, most firms continue to employ the previous generation of cybersecurity solutions [1, 2]. These new attacks can get past the static defenses that companies have already implemented. The fact that Americans spent more than $ 600 billion on e-commerce in one year raises serious questions about cyber security in today's world [3]. Security professionals struggle to safeguard this increasingly vital cyberspace [4, 5]. When applying security analytics, the issue of class imbalance is a crucial consideration for defenders [6]. The term “class imbalance” refers to how one class label is underrepresented while other classes have high representation [7].

Big data frameworks, such as Apache Spark and deep learning (DL) algorithms, are being implemented to improve the system's overall efficacy and scalability. The DL algorithms' CNN has proven to be quite valuable in computer vision, particularly for tasks such as recognizing people, digits, and various types of classification. The convolutional procedure extracts information from a predetermined kernel or window [8]. The LSTM is a type of recurrent neural network (RNN). The LSTM organizes its units using the gating concept. One of the main issues with RNNs is that they cannot learn about the environment for a long time; this is called the “vanishing gradient problem,” and it happens when there is no new data to train. As a result, RNNs cannot acquire knowledge from dependents at a great distance [9]. Utilizing an LSTM architecture is one approach that is considered to resolve this matter. It avoids the vanishing gradient problem and, as a result, makes it possible to hold on to the context information for a longer period. The Spark from Apache is one hundred times quicker than Hadoop [10]. Spark employs RDDs, and Hadoop employs map-reduce. Because network traffic is expanding quickly, a modern intrusion detection system (IDS) coupled with Apache, Hadoop, and Apache Spark is needed. One of our models used Apache Spark, a free source large data processing engine. Spark can analyze datasets on many nodes in parallel, prioritizing speed. It is 100 times faster than Hadoop and Storm [11].

The data must first be preprocessed, clearing the data of undesired values and redundant features. The proposed work needs to supply high-quality data for step processing the data when the data are analyzed to refine the data more effectively. The data standard is an essential factor in the outcomes. Preprocessing the data means solving the null values and deleting the features containing zero values after data preprocessing, selecting the most important features using random forest, which gave 19 from 84 features. Three models were proposed, two deep learning (CNN and LSTM) and Apache Spark. The Apache Spark model gave the best results for 15 classes. The accuracy and *F*1-score performance metrics are used to evaluate the three models for NIDS using the CSE-CIC-IDS2018 datasets.

The course of the present research work is motivated by providing supportive evidence for using the best techniques for detecting intrusions and attacks on Big Data. Furthermore, exploring different approaches used for addressing IDS over Big Data is one of the aspects of the present research. Moreover, the overall objective is to detect the intrusion from Big Data in NIDS with less time and high accuracy. As per the development in Internet technology and the rising number of network attacks, network ID has become an investigation issue.

The contributions of this work are as follows:

- It has reduced the dimensionality of the features from 84 to the most important 19 features
- It decreased the imbalanced data using oversampling and undersampling techniques to reduce the imbalance ratio
- To increase IDS performance, it built three models, Apache Spark, CNN, and LSTM
- This paper proposed the Apache Spark model, the fastest model compared to DL models and previous work, with only 7.56 minutes of training time

The remaining sections of the paper are structured as follows: Section 2 is a literature review on IDS attacks in a network environment.  Section 3 introduces the proposed system.  Section 4 deals with results and discussion, and Section 5 includes conclusion and future work.

## 2. Literature Review

Literature research's primary topic was the technology behind big data and IDS based on machine learning. The following section discusses the strategies utilized in some of the most advanced NIDS available today. These systems are developed with machine learning and big data technology.

None of the four preceding studies' [1, 12–14] class imbalance analysis produced any findings for attacks that were made using CSE-CIC-IDS2018. There is no application of sampling approaches to investigate class imbalance concerns regarding attacks in CSE-CIC-IDS2018. None of the four investigations examined CSE-CIC-typical IDS2018's traffic (overall days) in conjunction with the assaults. We can experiment with the class imbalance and big data difficulties by reducing the class imbalance. For each of the three distinct web attack categories (“Brute Force-Web,” “Brute Force-XSS,” and “SQL Injection”), three of the four studies [1, 12, 14] used multiclass classification for “Web” attacks, resulting in deficient classification performance. For aggregated web attacks, categorization results are exceptionally high.

Based on computing restrictions, Basnet et al. [12] used the CSE-CIC-IDS2018 and offered comprehensive findings. They classified cyberattacks with 99.9% accuracy. Due to class imbalance, 99.9% accuracy is feasible even with zero positive class classifications in aggregated online attacks. High-class imbalance requires more sensitive performance metrics. Basnet et al. deleted twenty thousand samples with “Infinity,” “NaN,” and missing values from CSE-CIC-IDS2018. Except for the destination port and protocol, other features are numeric. Eight fields with zero values are among their 79 cleansed features. These zero-filled fields should have been filtered out. None of the eight features that have zero values indicated were filtered out.

Atefinia and Ahmadi [1] suggest a “modular deep neural network model” using CSE-CIC-IDS2018 data. Their multiclass classification was terrible. Only one customized learner makes benchmarking challenging. Atefinia and Ahmadi's guidelines are ambiguous. According to Atefinia and Ahmadi, online attacks combine the two attack days with typical traffic for only those two days. The other three CSE-CIC-IDS2018 experiments investigate these two days individually. Their new model misclassifies Web attacks and ignores class imbalance. Atefinia and Ahmadi provide no preprocessing data other than mentioning that missing rows and columns are removed. This comment is ambiguous because they could have mentioned the missing columns. A small portion of CSE-CIC-IDS2018 contains only 59 instances of null values infinity, and NaN is not recognized.

Ferrag et al. [15] compared multiple deep learning models and network intrusion datasets available to researchers. They provide an explanation of each of the different DL approaches as well as a description of 35 different datasets. They then performed their experiments using two of the listed datasets and seven models, including supervised and unsupervised learners. Their experiments were run using the Bot-IoT dataset which is high, with each model performing at around 98% accuracy. The models included are RNN, DNN, restricted Boltzmann machine (RBM), CNN, deep Boltzmann machines (DBM), and DAE.

Dwibedi et al. [16] analyzed and contrasted several recently released intrusion detection datasets. Statistical analysis focused on the distribution of protocols and attacks and compared performance analysis. They used various ML models, both classical and deep learning, to evaluate performance. SVM, RF, XGBoost, and Keras were among the models used. To develop and validate the models, we used several datasets. Experimentally, all models scored exceptionally well, except for Keras, which came in last with a precision and recall rate of only 97%.

Basumallik et al. [17] used convolutional neural networks to detect packet-data anomalies. Event signatures are extracted using a convolutional neural network filter (features). Two different bus systems are used to build the phasor measurement unit buses. The study found that an accuracy of 98.67% can be reached. They claim that the convolutional neural network-based filter outperforms other machine learning approaches such as RNN, LSTM, SVM, and bagged and boosted approaches.

Fu et al. [18] created a framework that uses a convolutional neural network to capture the inherent patterns of fraud behaviour to detect credit card fraud. Zhang et al. [19] trained and tested a convolutional neural network using the data. A month's worth of data was split into training and testing sets. The study claims a 91% accuracy rate and a 94% recall rate. In comparison with Fu et al. [18], these findings have been improved by 26% and 2%, respectively.

Nasr et al. [20] introduced a convolutional neural network called DeepCorr for intrusion detection. A neural network with three linked layers and two convolutional layers is used to build DeepCorr. According to an experiment, DeepCorr performs best with a learning rate of 0.0001 while utilizing a false positive rate of 10–3.

Zhang et al. [21] created an anomaly traffic identification model based on two layers of neural networks. The first layer is an updated version of the LetNet-5 convolutional neural network. In the second layer, we make use of our long-term memory. The flow will first have its features connected to space and time extracted, and then the second layer's features will have their time-related characteristics extracted.

## 3. Proposed System

It is explained in this section how NIDS's deep learning and Apache Spark are designed. The CSE-CIC-IDS2018 dataset, a real-world network traffic dataset, is used in this section for NIDS implementation. The ideal technique to test and evaluate a network's applications and lowest-level entities is through Amazon (AWS) real-time traffic, a transition from static to dynamic data [21].

**Figure 1:**
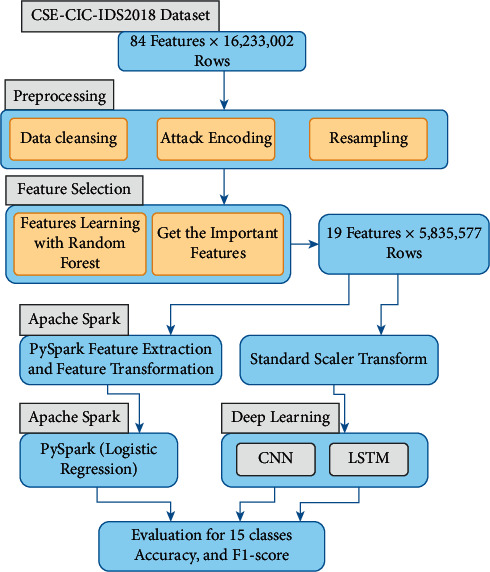
Framework for the proposed system.

All zero-valued features were removed from the CSE-CIC-IDS2018 dataset and converted each class to a numeric value. The main processes in the proposed network intrusion detection system are data preprocessing, feature selection, and three models (CNN, LSTM, and Apache Spark).  Figure 1 shows the proposed system framework.

### 3.1. Description of the Dataset

This analysis will use the Canadian Institute of Cybersecurity's 2018 CSE-CIC-IDS2018 dataset. The AWS CLI is a command-line interface for Amazon Web Services that can be used to get the PCAP, CSV, and log files from an Amazon web server [22]. This study relied on CSV files. It contains 6.89 GB of data spread out among 10 CSV files. It is possible to find other intrusion datasets such as DARPA98, KDD99, ISC2012, and ADFA13 [23]. However, the attacks detailed in these datasets do not apply to the current period. CSE-CIC-IDS2018 is a recent dataset with 84 features and 16.23 million rows of information.

For each category, the dataset includes fourteen known and current attacks. Having the most recent and up-to-date information is critical when dealing with attacks. The dataset comprises fourteen labels based on the approaches used. DoS, distributed denial of service (DDoS), brute force, botnet, infiltration, and web-based attacks are among the six groups of attacks used.

The recent dataset includes fourteen attacks, as shown in Table 1. Raw data were recorded and edited daily. When creating data, more than 80 statistical features are calculated in the forward and reverse directions, and information is given on whether an attack is added. The final dataset is published online, with over 16 million records spread out among ten files in PCAP and CSV data.

### 3.2. Data Preprocessing

Cleaning data of redundant values and properties are data preparation goals. The data analysis phase will be more efficient if it offers high-quality data. The results depend heavily on the preprocessing of the data. Preprocessing removes features with zero values, solves null values, and prepares the data for manipulation. The following are some measures to follow to achieve the preprocessing section goal:

- Cleansing data means eliminating errors, omissions, and inconsistencies [24, 25]. Python was the programming language of choice for our project. Eight features were removed from the dataset after it was determined that it had no data. The dataset had object, float, and integer as data types. It should be in float to increase its usability and prepare it for algorithms. Infinity and NaN values were transformed using the median.
- Attack encoding: the attack encoding approach is a popular choice when dealing with categorical variables. Each label is assigned a unique integer in this method. The dataset has been labelled with fifteen different types of attacks, fourteen attacks, and benign. To get it ready for machine learning, it is numbered from 0 to 14.
- Class imbalance on dataset: class imbalance is the number of occurrences in which classes are out of balance. Many classification issues with real-world data show an imbalance in the number of classes. The benign class has the most samples in the CSE-CIC-IDS2018 dataset, which has a class imbalance. This huge mismatch makes classification challenging. Misclassification of the minority class is common when datasets are unbalanced, which causes a bias in classification towards the majority class. The class imbalance has been solved using an oversampling technique. Oversampling and undersampling techniques are part of this strategy. There are a variety of tools available for resampling datasets. Adding random instances from the minority class to the dataset is known as random oversampling.
- Random undersampling (RUS): this sampling method removes instances from the majority class to improve class imbalances toward the desired target classes. In [26, 27], RUS is more successful than other sampling methods. Additionally, RUS has been used in other studies [28, 29] to address the issue of class imbalance.

CSE-CIC-IDS2018 has a colossal imbalance because the benign class was 13484708, and the smallest class was only 87. After resampling, as shown in Table 1, variances between classes have been reduced. The variance between class 13 and class 0 was (87/13,484,708), which equals 0.0000006, but after resampling and undersampling, it is (30,000/300,000), which equals 0.1. Table 1 illustrates the number of samples before and after notice; only classes 2, 3, 5, and 13 had upsampling, and class 0 had undersampling. Attack names and the number of samples before sampling in Table 1 are available in the “Label” column in the dataset [23].

### 3.3. Feature Selection

It is necessary to consider the varied importance of each feature to determine the essential features [30]. In some cases, feature selection can advance the model's understanding of the solved problem [31, 32]. For selecting the features, the study used Scikit learn and RF to get the essential features. Features are given importance in the random forest algorithm. An IDS intended for big data networks with high volume and velocity must use a feature selection mechanism. The random forest (RF) classifier methods are used to choose the features in this research. Experiments have shown that the feature set picked by our suggested RF approach appears promising and suited for large-scale network IDSs, as demonstrated by the findings.

**Figure 2:**
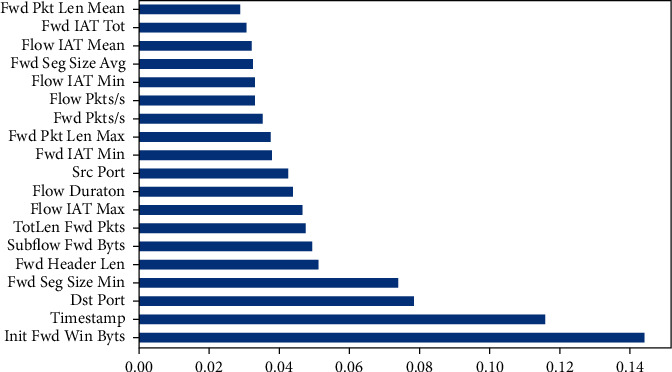
Important features by random forest.

The important features are selected using the RF. The dataset splits into 70% for training and 30% for testing with random_state = 42, and n_estimators = 1000. The training time took 64.12 minutes. Then, the selector from the random forest classifier model identifies the features that have importance more than the 0.20 threshold. The training time of the selector took 64.9 minutes. The selector model gave 19 features, as shown in Figure 2.

### 3.4. Implementation Environment

In the proposed study, we used Anaconda 3, the Python 3 distribution, Jupyter Notebook, the Sklearn Python Machine learning RF, deep learning CNN and LSTM, and Python Pyspark to eliminate unnecessary features, reduce the dataset's dimension, and build the three models. The proposed models are implemented in Python programming (Anaconda and Jupyter) using the machine learning RF algorithm and deep learning MLP algorithm. Tests were conducted on a personal computer with a 2.6 GHz Core (TM) i7-10750H CPU and 32 GB of memory under Windows10.

### 3.5. Models

When selecting neural networks, it is crucial to consider the features' characteristics. This study benefited from Apache Spark logistic regression, CNN, and LSTM because the traffic data are arranged as a sequence. Keras, which is built on the top of Tensorflow, is used to construct deep learning models and is then used to apply deep learning models [33]. The study proposed three models, one using Apache Spark and for supervised deep learning using CNN and LSTM.

#### 3.5.1. Apache Spark

**Figure 3:**
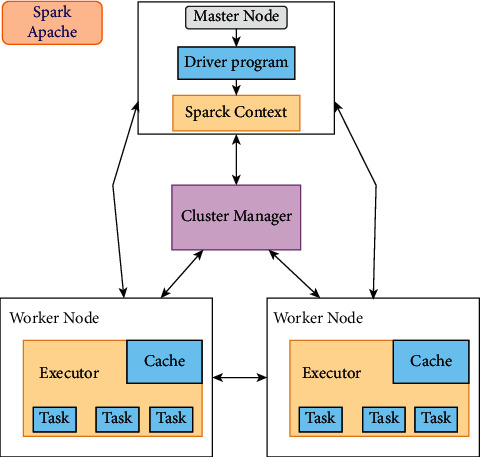
Framework of Spark.

It is a cluster computing platform built primarily to process massive amounts of data. It is an open-source project. It uses a multistaged in-memory processing technique that results in processing that is 100 times quicker than map-reduce processing. Spark can manage Hadoop clusters, access and analyze any Hadoop data source, and run Hadoop jobs. The core of Apache Spark is where some of the program's most fundamental capabilities are housed [34]. The use of logistic regression as a technique for predicting a categorical response is quite common. It is a subtype of a generalized linear model that can predict the likelihood of various events. You can use ML logistic regression in Spark to predict a binary outcome by employing binomial logistic regression, or you can use it to predict a multiclass outcome by employing multinomial logistic regression. Both of these methods are described in the following paragraphs. The logistic regression multinomial method is utilized for multiclassification purposes on Apache Spark.  Figure 3 illustrates the framework of Apache Spark.

#### 3.5.2. Convolutional Neural Network (CNN)

It is a sophisticated model implemented in various contexts. An operation that combines two processes that are happening at the same time is called a continuous convolution function [24, 35]. A comparison is made between the CNN model based on feature sequencing and the existing convolutional neural network. The input layer, the convolution layer, the pooling layer, the full connection layer, and the output layer are the components that make up the CNN, which is a feedforward neural network [36]. In the same way as conventional neural networks, it is made up of neurons with weights that can be taught and bias constants that can be adjusted. In the convolution layer, the implicit unit is the only one that can connect a portion of the input unit; it cannot connect all of the input units. The conventional CNN alternates the convolution layer and the pooling layer. The number of convolution layers and activation functions of CNNs with varied topologies is also different [37, 38].

#### 3.5.3. Long Short-Term Memory (LSTM)

As proposed by Hochreiter and Schmidhuber [39], it is ideally suited for learning from experience and classifying time series. The LSTM recurrent neural network structure was made to deal with the problem of long-term dependence. It adds a gate for forgetting, input, and output to the conventional Recurrent Neural Networks (RNN) [40]. When a neural network forgets the knowledge, it does not need, the input gate sends new information to the network, and the output gate decides what happens to that information [41].

## 4. Results and Discussion

Feature reduction, also known as dimensionality reduction, decreases the number of features in a resource-intensive computation without losing vital information. Following feature selection by RF, the resulting dataset had only the 19 most significant features and 5,835,771 rows, totalling 5,835,771. Figure 2 illustrates the importance of the chosen features. According toTable 2 and Figure 2, our proposed models achieve the best reduction in feature count from 84 to 19 features.

In this experiment, the hyperparameters for CNN and LSTM are hidden nodes “200,” loss “categorical_crossentropy,” batch size “128,” and initial learning rate “0.0001,” activation function “Softmax,” and optimizer “Adam.” Training will proceed slowly if the learning rate is too low since only a few updates will be to the network's weights. The dataset splits into 70% for training and 30% for testing.

This study used accuracy and *F*1-score to evaluate the performance of the models. The accuracy of the Spark model is 100%, as Table 3 illustrates the accuracy of the three models; moreover, the *F*1-score is the best in the Apache Spark model; it gave 1 for most classes. For the LSTM and CNN models, the accuracy and *F*1-score are high for most classes, as shown in Tables 2 and 3.

### 4.1. The Performance Plots of the Deep Learning Models

**Figure 4:**
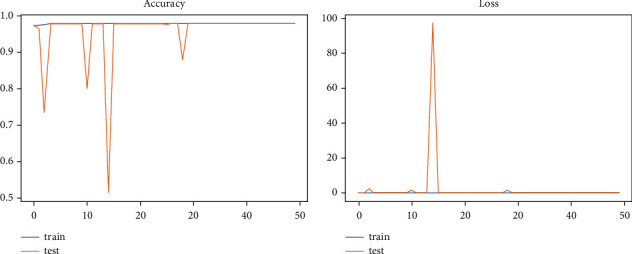
CNN performance plots.

**Figure 5:**
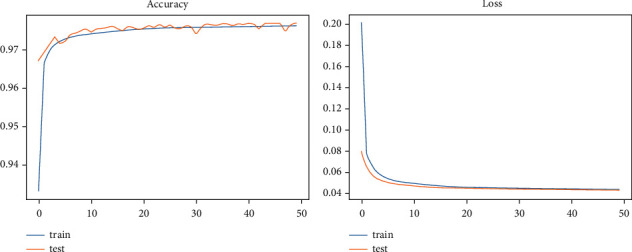
LSTM performance plots.

Model accuracy and log loss are computed for each epoch's training and testing sets. It helps to determine whether the model has been successfully trained to classify various attack types and how many samples in the testing set have been accurately classified. The loss represents the total number of mistakes for each training sample. In the study of the log loss (categorical_crossentropy) for the DL models, it is evident from the loss plot that the testing set has extremely few incorrect predictions. To correctly train the models, the number of epoch values is 50 for this experiment phase due to the dataset size. Figures 4 and 5 demonstrate that the suggested models can categorize various types of intrusions in the selected datasets.

**Figure 6:**
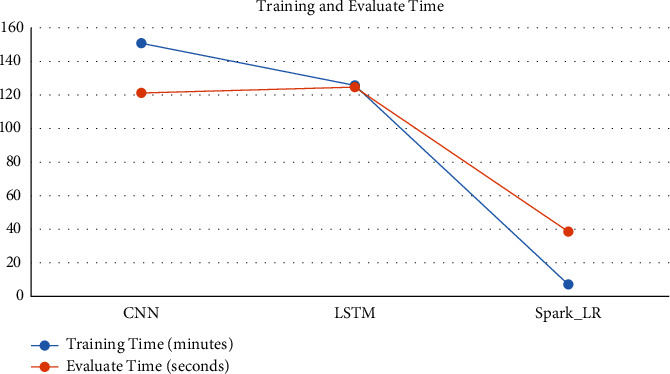
Time of training and evaluation.

The Apache Spark model gave the best result for all classes as multiclassification; furthermore, it took the least time for training and evaluating, only 7.56 minutes for training and 39 seconds for evaluating, while the LSTM model took 125.29 minutes for training and 124.65 seconds for evaluating. CNN took 150.26 minutes and 120.65 seconds to evaluate, as shown in Figure 6.

The Apache Spark model is the fastest if compared with all previous works. Moreover, the Apache Spark model has the best result with 100% accuracy for all classes and the best results for the *F*1-score, as shown in Tables 2 and 3. Furthermore, our proposed models used the RF method to reduce the dimensionality of features, the best feature set for large-scale NIDS. RF gave the most important features and reduced the features from 84 to only 19, as shown in Figure 2.

The three models are multiclassification for detecting 14 attacks and benign if comparing the results of the proposed models to the deep learning and Apache Spark literature works. The three models gave the best results (accuracy and *F*1-score), especially the Apache Spark model. Furthermore, the three models are multiclassification for detecting 14 attacks and benign. Moreover, the proposed work reduces dimensionality from 84 features to only 19, with the best result (accuracy and *F*1-score). The Apache Spark model is faster than deep learning and models of all previous works.

### 4.2. Confusion Matrix

In contrast to binary categorization, neither positive nor negative classifications exist here. Since there are no positive or negative classes, it may initially be challenging to determine TP, TN, FP, and FN. Start determining TP, TN, FP, and FN for each class. Then, let us check the values of the confusion matrix's metrics.

**Figure 7:**
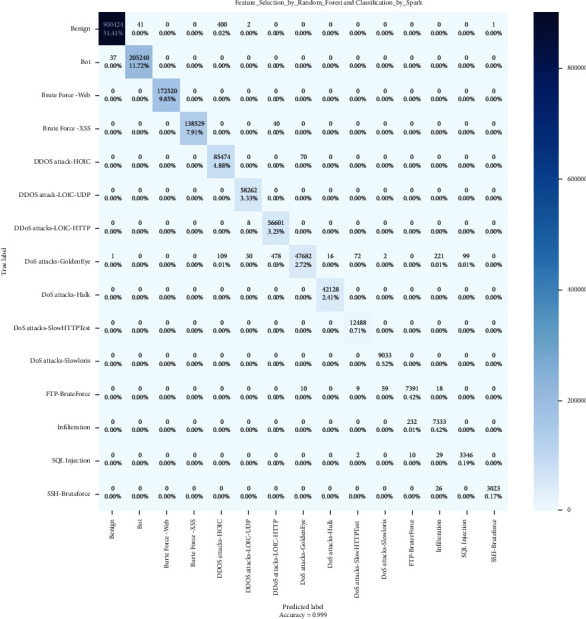
Spark confusion matrix.

**Figure 8:**
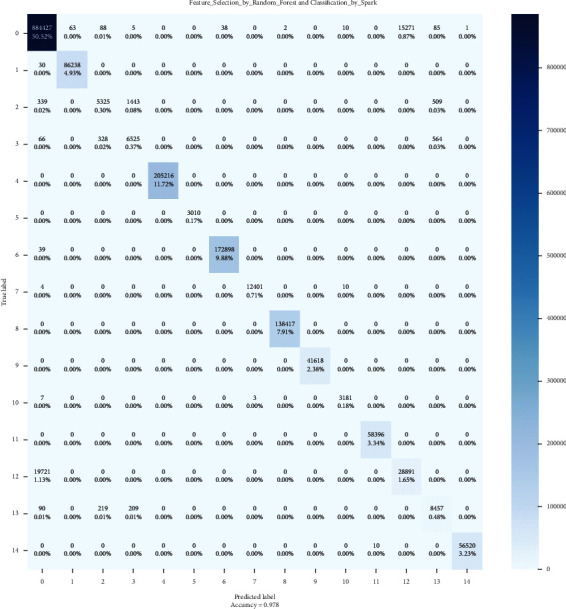
CNN confusion matrix.

**Figure 9:**
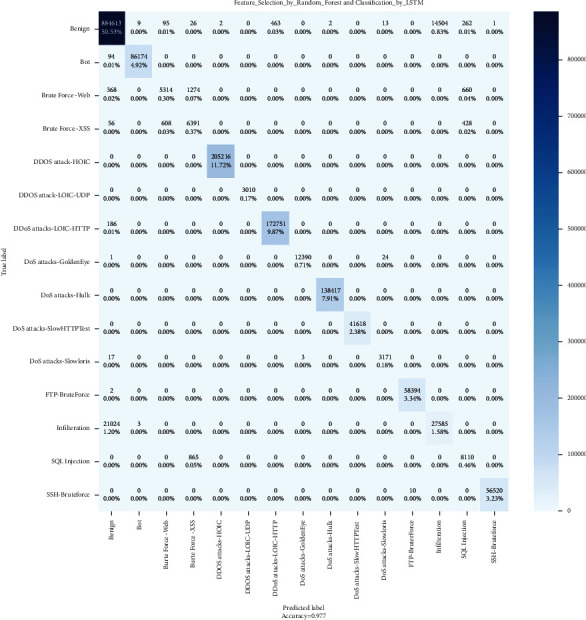
LSTM confusion matrix.

The confusion matrix technique is typically utilized to solve classification problems because it accurately represents the actual and predicted classes' output. Using resemblance measures determines the pairwise similarity between every class set. Figures 7–9 show the confusion matrixes.

## 5. Conclusion

This study proposed three models, Apache Spark, CNN, and LSTM, to detect network attacks. The proposed models reduced the dimensionality for feature reduction from 84 to 19 using the random forest algorithm. The following studies with fourteen sorts of attacks, and the introduced work focuses on the three models for categorizing attacks. The dataset is imbalanced; therefore, oversampling and undersampling are employed to lower the imbalance ratio. Accuracy and *F*1-score are used to compare the detection ability of these three models.

Moreover, Apache Spark gave 100% accuracy for all classes and *F*1-score 1 for ten classes and 0.99, 0.98, 0.97, and 0.98 for the remaining classes. The Apache Spark is the fastest model compared to DL models and previous related work, with only 7.56 minutes of training time. Accordingly, there are limitations to the study; it only detects the existing signature-based intrusion detection systems. Thus, we suggest important future work, including semisupervised learning.

## Figures and Tables

**Table 1:** Attacks samples before and after resampling.

Class no.	Attack name	No. of samples before resampling	No. of samples after resampling
0	Benign	13,484,708	3,000,000
1	Bot	286,191	286,191
2	Brute Force-Web	611	25,000
3	Brute Force-XSS	230	25,000
4	DDOS attack-HOIC	686,012	686,012
5	DDOS attack-LOIC-UDP	1,730	10,000
6	DDoS attacks-LOIC-HTTP	576,191	576,191
7	DoS attack-GoldenEye	41,508	41,508
8	DoS attack-Hulk	461,912	461,912
9	DoS attack-SlowHTTPTest	139,890	139,890
10	DoS attack-Slowloris	10,990	10,990
11	FTP-Brute Force	193,360	193,360
12	Infilteration	161,934	161,934
13	SQL Injection	87	30,000
14	SSH-Brute Force	187,589	187,589
Total		16,232,943	5,835,577

**Table 2:** Performance of the three models (*F*1-score).

*F*1-score			
Class	CNN	LSTM	Spark_LR
0	0.98	0.98	1.00
1	1.00	1.00	1.00
2	0.78	0.78	1.00
3	0.83	0.80	1.00
4	1.00	1.00	1.00
5	1.00	1.00	1.00
6	1.00	1.00	1.00
7	1.00	1.00	0.99
8	1.00	1.00	1.00
9	1.00	1.00	1.00
10	1.00	0.99	1.00
11	1.00	1.00	0.98
12	0.62	0.61	0.97
13	0.91	0.88	0.98
14	1.00	1.00	1.00

**Table 3:** Performance of the three models (accuracy).

Accuracy			
Class	CNN	LSTM	Spark_LR
0	0.98	0.98	1.00
1	1.00	1.00	1.00
2	1.00	1.00	1.00
3	1.00	1.00	1.00
4	1.00	1.00	1.00
5	1.00	1.00	1.00
6	1.00	1.00	1.00
7	1.00	1.00	1.00
8	1.00	1.00	1.00
9	1.00	1.00	1.00
10	1.00	1.00	1.00
11	1.00	1.00	1.00
12	0.98	0.98	1.00
13	1.00	1.00	1.00
14	1.00	1.00	1.00

## Data Availability

The article includes the tables and figures that support this study's findings.
